# Biophysical characterization of Ca_V_1.4 L-type calcium channel mutants causing congenital stationary night blindness type 2 in humans

**DOI:** 10.1186/2050-6511-13-S1-A69

**Published:** 2012-09-17

**Authors:** Klaus W Schicker, Verena Burtscher, Dagmar Knoflach, Anamika Singh, Thomas Stockner, Alexandra Koschak

**Affiliations:** 1 Department of Neurophysiology and Neuropharmacology, Center for Physiology and Pharmacology, Medical University of Vienna, 1090 Vienna, Austria; 2 Department of Pharmacology and Toxicology, Institute of Pharmacy, University of Innsbruck, 6020 Innsbruck, Austria

## Background

Ca_V_1.4 L-type calcium channels show unique biophysical properties such as slow inactivation due to the lack of calcium-dependent inactivation (CDI). These properties make Ca_V_1.4 channels appropriate candidates for triggering persistent glutamate release at retinal photoreceptor cell synapses. Mutations in the CACNA1F gene encoding for the Ca_V_1.4 α1 subunit are described in patients with X-linked congenital stationary night blindness type 2 (CSNB2). Impaired transmission between rod photoreceptor cells and second-order neurons manifests as night blindness and various other visual symptoms in the affected individuals.

## Methods

The aim of this study was to investigate the functional properties of Ca_V_1.4 mutants L849P and R1816stop compared to wild-type (WT) in transiently transfected tsA 201 cells (+β3,+α2δ-1) via whole-cell patch clamp technique using 15 mM Ba^2+^ and Ca^2+^ as charge carrier. For statistics, either Mann-Whitney (two groups) or Kruskal-Wallis test and Dunn’s Post hoc test (multiple comparisons) were used.

## Results

L849P was mainly characterized by a reduced current density (pA/pF: WT: −16.3 ± 1.6 (n = 38), L849P: −2.5 ± 0.3 (n = 12), p < 0.0001; Ca^2+^), only minor, not significant (p > 0.05) changes in the voltage-dependent activation properties were observed. In presence of the dihydropyridin-activator BayK8644 (5 μM) the current density was increased ~10-fold (p < 0.001). The fold-increase in current density was comparable to WT. As expected R1816stop, which lacks an intrinsic C-terminal modulator (CTM), exhibited CDI (f-value: WT: 0.11 ± 0.03 (n = 8); R1816stop: 0.63 ± 0.02 (n = 22)) and shifted the voltage-dependence of activation to more negative voltages (V0.5_act_ in mV: WT: 1.8 ± 0.3 (n = 74), R1816stop: −12.3 ± 0.3 (n = 23)). In presence of the Ca_V_1.4-CTM; comprising the last 122 C-terminal residues WT conditions were fully restored, e.g. V0.5_act_ 2.2 ± 1.0 mV (n = 14) (p < 0.0001).

## Conclusions

We assume that the reduced current density observed in mutant L849P derives from decreased channel expression, which might be explained by a folding defect of the Ca_V_1.4 channel protein rather than a reduced open probability. Moreover, the fact that the functional phenotype of the R1816stop can be rescued bears a potential pharmacotherapeutic concept based to the C-terminal modulatory mechanism present in Ca_V_1.4 channels.

